# Application of indocyanine green-mediated fluorescence molecular imaging technology in liver tumors resection: a systematic review and meta-analysis

**DOI:** 10.3389/fonc.2023.1167536

**Published:** 2023-06-13

**Authors:** Gang Zhu, Xing Qiu, Longfei Zeng, Zhirui Zou, Liu Yang, Shanmao Nie, Zuanyu Wang, Xin Zhang, Jinquan Tang, Yong Pan, Shaozhen Tang, Tao Wu

**Affiliations:** ^1^ Department of Hepatobiliary Pancreatic and Splenic Surgery, Luzhou People’s Hospital, Luzhou, China; ^2^ Department of Gastroenterology Medicine, Luzhou People’s Hospital, Luzhou, China

**Keywords:** indocyanine green, fluorescence molecular imaging, liver tumors, hepatectomy, meta-analysis

## Abstract

**Background:**

This meta-analysis was dedicated to evaluating the safety and effectiveness of indocyanine green (ICG) -mediated fluorescence molecular imaging (FMI) technology in liver tumors resection.

**Methods:**

A literature search of PubMed, Embase databases, Cochrane Library, and Web of Science was performed to identify all clinical controlled studies exploring the effects of fluorescence imaging on liver tumors resection. Quality assessment and data extraction of studies were conducted independently by 3 reviewers. Mean difference (MD) and odds ratio (OR) with 95% confidence interval (CI) were calculated using a fixed-effects or random-effects model. The meta-analysis was performed with RevMan 5.3 software.

**Results:**

14 retrospective cohort studies (RCSs) involving a total of 1227 patients were finally included. The results showed that Fluorescence-assisted liver tumors resection could improve the R0 resection rate (OR = 2.63; 95% CI: 1.46~4.73, *p* = 0.001), reduce overall complications (OR = 0.66; 95% CI: 0.44~0.97, *p* = 0.04), biliary fistula (OR = 0.20; 95% CI: 0.05~0.77, *p* = 0.02), intraoperative blood loss (MD = −70.76, 95% CI: −106.11 to −35.41; *p* < 0.0001), and shortens hospital stay (MD = −1.41, 95% CI: −1.90 to −0.92; *p* < 0.00001). There were no significant differences in the incidences of operative time (MD = −8.68, 95% CI: −18.59 to −1.22; *p* = 0.09), complications of grade III or above (OR = 0.73; 95% CI: 0.43~1.25, *p* = 0.26), liver failure (OR = 0.86; 95% CI: 0.39~1.89, *p* = 0.71), and blood transfusion (OR = 0.66; 95% CI: 0.42~1.03, *p* = 0.07).

**Conclusion:**

Current evidence suggests that ICG-mediated FMI technology could enhance the clinical effectiveness of patients with liver tumors resection and is clinically worthy of promotion.

**Systematic review registration:**

PROSPERO, identifier CRD42022368387.

## 1 Introduction

Hepatectomy is the most effective treatment for liver tumors (1–3). Intrahepatic recurrence rates after resection of liver tumors remains high, despite improvements in preoperative imaging modalities and chemotherapy regimens (4, 5). In addition, some the patients develop recurrence within 12 months, suggesting that tumors were missed previously (6). The patient’s long-term findings are directly related to the complete resection of all tumor cells under edge microscopy, which is, the surgical resection “R0” pursued by all surgeons. However, in fact, current preoperative imaging techniques such as improved computed tomography (CT) or nuclear magnetic resonance imaging (MRI) have a lower sensitivity to millimetric-sized liver lesions (7–9). During surgery, visual appearance, palpation, and intra-operative ultrasound results are the basic means of determining if tumor-free margins are obtained (10, 11), and these are difficult to find small and/or deep lesions, or to identify these lesions with dysplastic nodules. Therefore, a novel diagnostic tool is required to detect such potentially neglected small liver tumors and guide the operation in real-time.

Indocyanine green (ICG) has been widely used as a diagnostic agent to measure patients’ preoperative liver function to prevent post-operative liver failure (12). ICG binds to proteins in the blood and is then rapidly taken up by hepatocytes, thus quickly disappearing from the bloodstream (half-life ~ 3-4 minutes). In addition, ICG can accumulate in specific areas and emit fluorescent signals at near-infrared (NIR) wavelengths (13, 14). In summary of these two features, it has become a good NIR fluorescent probe for solid tumors. The mechanism of the specific accumulation of ICG in liver tumors is not “unclear”, studies have shown that preserved portal uptake of ICG in differentiated hepatocellular carcinoma (HCC) cells by transporter proteins NTCP and OATP8 with concomitant biliary excretion disorders causes accumulation of ICG in the cancerous tissues (15). All in all, the accumulation of ICG in liver tumors is not without evidence.

Since Ishizawa et al. (16) first used ICG-mediated fluorescence molecular imaging (FMI) technology to clearly show colorectal liver metastasis (CLM) and HCC, similar studies have been reported successively, however, most of them are single-arm studies or small sample single-center clinical controlled studies. There remains a lack of strong clinical evidence on whether an ICG-mediated FMI can improve the effectiveness of liver resection. In this article, we have incorporated the latest published controlled clinical studies to explore the safety and efficiency of the FMI in resection of liver tumors.

## 2 Materials and methods

This meta-analysis has been reported in line with the Preferred Reporting Items for Systematic Reviews and Meta-Analyses Statement and Assessing the Methodological Quality of Systematic Reviews Guidelines (17). And the prospective protocol for this study was registered with the PROSPERO (registration number: CRD42022368387).

### 2.1 Literature search

Systematic retrieval of relevant English documents since the establishment of PubMed, Embase, Cochrane Library, and Web of Science, to find documents that are in line with the research. These studies compared fluorescent imaging-guided liver tumors resection with non-fluorescent imaging-guided liver tumors resection. Keywords retrieved include: indocyanine green fluorescence, ICG fluorescence, liver resection, hepatectomy, hepatectomies, liver neoplasm, hepatic neoplasm, hepatic cancer, liver cancer, hepatocellular cancer, hepatocellular carcinoma, cancer of liver, liver tumor, and liver metastases. Re-search according to the references of the documents obtained from the search to improve the detection rate of qualified documents.

### 2.2 Inclusion and exclusion criteria

The inclusion criteria were as follows: (1) Patients: Patients of any age, gender, and race have undergone hepatoma resection (2) Intervention method: The FMI group performed liver tumor resection under the guidance of fluorescence, and the Conventional group performed tumor resection under the guidance of non-fluorescence. (3) At least one outcome has been reported in the literature: operative time, R0 resection rate, overall complications, complications of grade III or above, biliary fistula, liver failure, intra-operative blood loss, blood transfusion, and hospital stay. The exclusion criteria were as follows: (1) studies without a control group; (2) case reports, letters, reviews, conference reports, or experiments; (3) conference abstracts without the full text; (4) The sample size in each group is less than 10.

When publishing research with repeated experimental data, the latest publication will be selected and previous articles will be used to supplement the necessary information in the new research.

### 2.3 Study selection and data extraction

All studies are independently and systematically reviewed by three authors (ZW, XZ, and JT). The included studies were then blindly and independently extracted and recorded by the above three authors and recorded the following data: first author, country, sample size, clinical traits of patients, histology, tumor diameter, and liver function, The authors were contacted *via* e-mail to obtain any missing information. Patients were divided into FMI group and Conventional group according to different treatment methods, and differences were resolved through negotiation. For quantitative data without means or standard deviation (SD), the mean and SD are estimated based on the median, range/quartile, and sample size (18, 19).

### 2.4 Methodological quality assessment

Use the Newcastle-Ottawa Scale (NOS) to assess the methodological quality of the included studies (20). The maximum score on the scale was 9, and studies with scores >5 were considered to have high methodological quality. Disagreements were resolved by common consensus.

### 2.5 Statistical analyses

The Cochrane Review Manager software (RevMan; version 5.3) was used to combine the study-specific odds ratio (OR) of categorical variables, the continuously varying mean difference and its 95% confidence interval (CI) to calculate the combined value of each study. Heterogeneity was studied using *χ^2^* text and *I^2^* statistics. If *I^2^* ≤ 50.0% or *p* ≥ 0.1, no heterogeneity occurs, so the fixed -effects model was used to merge the data. On the contrary, the random-effects model was performed. By deleting one study at a time and repeating the meta-analysis to evaluate whether any one study has a significant impact on the combined estimated value, so as to conduct a sensitivity analysis. Potential publication bias was assessed by visually inspecting the funnel plots.

## 3 Results

### 3.1 Search results, characteristics, and quality of included studies

**Figure 1:**
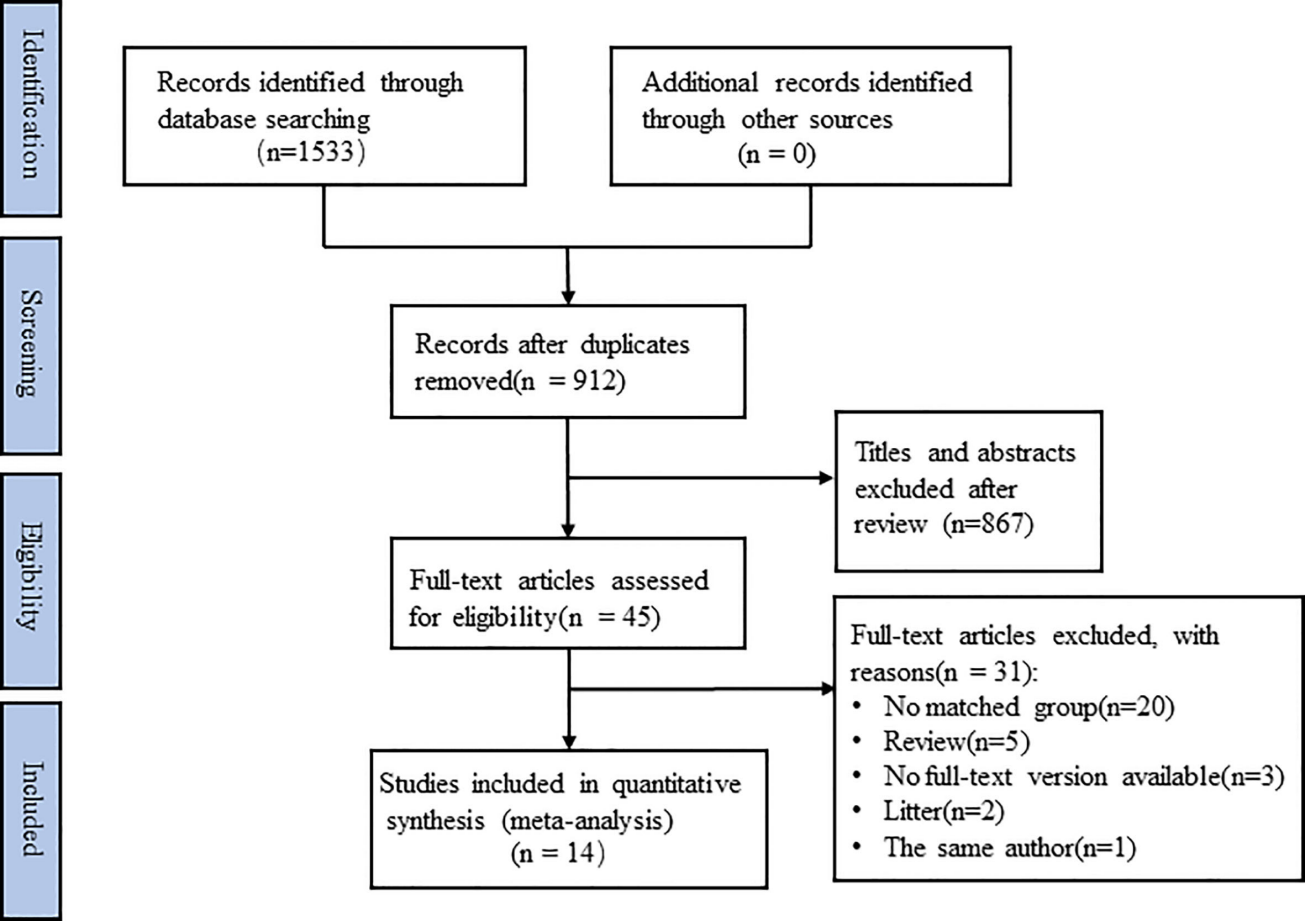
Flow diagram of literature selection.

**Figure 1** shows the search strategy of the research. 1533 studies were initially retrieved, of which 912 studies were removed due to duplication, and 867 studies were removed after reading the title and abstract. The remaining 45 studies were screened through careful reading of the full text, and 31 studies were excluded for the following reasons: no matched group (n = 20), review (n = 5), no full-text version available (n = 3), litter (n = 2) and the same author (n = 1). Finally, 14 retrospective cohort studies (RCSs) were used in meta-analysis, involving 1227 patients, including 513 patients in FMI group and 714 patients in Conventional group. The characteristics of each included study are displayed in **Table 1**.

**Table 1:** Characteristics of the included studies.

References	Country	Age (years)	Sample Size		Imaging purpose	surgery type	Histology	Tumor Diameter (cm)	Child–Pugh (A)%	NOS
			FMI	C						
Aoki 2018 (21)	Japan	63 (34-84)/69(35-86)	25	72	TL	Lap	HCC/MLT	2.9 (0.8–5.7)/2.5 (0.7–4.8)		7
Chen 2022 (22)	China	57.3 ± 9.7/ 56.3 ± 12.1	48	60	TL	Lap	PLC	4.6 ± 2.5/5.3 ± 2.7	75/76.6	8
Cheung 2018 (23)	China	60.5 (47.0-7 3.0)/61.5(25.0-86.0)	20	120	TL	Lap/Open	HCC	2.75 (1.2–6.5)/3.45 (1–9.5)	100/100	8
Handgraaf 2018 (24)	Netherlands	62.0 ± 9.2/63.0 ± 9.4	67	87	TL	Open/Lap	MLT	NA		9
Itoh 2022 (25)	Japan	67 (44– 83)/69 (44– 87)	32	32	TL	Lap	HCC/ICC/MLT	1.9 (1.0–5.3)/2.0 (0.7– 4.3)	96.9/100	8
Kaibori 2011 (26)	Japan	70.4 ± 5.5/ 68.2 ± 11.0	52	50	CG	Open	HCC/ICC/MLT/BD	(5.3 ± 4.9)/ (5.0 ± 4.6)	94.2/96	8
Lu 2021 (27)	China	57.3 ± 12.2/55.2 ± 12.5	57	63	HS	Lap	PLC/BD	4.5 (2.5–5.1)/4.5 (3.2–6.5)		7
Marino 2019 (28)	Italy	66.2 (35–78)/64.6 (33–77)	25	25	HS	Robot	HCC/ICC/MLT	2.35 (0.2–4.7)/2.39 (0.5–5.2)	80/80	8
Nishino 2017 (29)	Japan	66.9 (39-82) /65.1 (33-82)	23	29	HS	Open	HCC/ICC/MLT/BD	NA	95.7/93.1	9
Wang^(1)^ 2022 (30)	China	NA	81	81	TL	Lap	HCC	3.5(2.3-5.4)/3.0(2.2-5.05)	100/100	9
Wang 2022 (31)	China	54.5 (46–60)/56 (45–70)	14	11	TL	Lap	NT	NA	100/91	8
Yao2020 (32)	China	52.9 ± 12.1/59.3 ± 10.4	18	29	HS	Open	HCC	6.3 ± 3.2/7.8 ± 2.5	NA	8
Zhang 2020 (33)	China	55.7 ± 11.2/52.5 ± 12.1	30	34	HS	Lap	HCC/ICC/BD	NA	90/100	8
Zhou 2019 (34)	China	NA	21	21	TL	Lap	HCC	3.1 (1.8-5.5)/3.2 (1.2-5.5)	85.7/85.7	8

FMI, FMI group; C, Conventional group; TL, Tumor localization; HS, Hepatic segmentation; CG, Cholangiography (detection of bile leak); HCC, Hepatocellular carcinoma; ICC, Intrahepatic cholangiocarcinoma; PLC, Primary liver cancer; MLT, Metastatic liver tumor; BD, Benign disease; NT, Neuroendocrine tumor; NA, Not available.

### 3.2 Meta-analysis outcomes

#### 3.2.1 Meta-analysis of operative time

**Figure 2:**
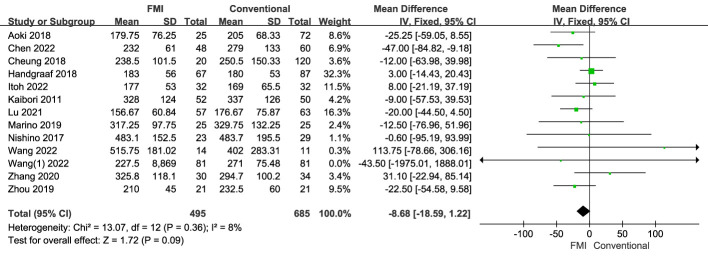
Forest plot on operative time.

13 studies (21–34) including 1180 patients reported the operative time, there was no heterogeneity between the two groups (*p* = 0.36, *I^2^ = *8%), and fixed-effects model analysis was used. The results of meta-analysis showed that there was no significant difference in the operative time between the two groups (MD = −8.68, 95% CI: −18.59 to −1.22; *p* = 0.09) (**Figure 2**).

#### 3.2.2 Meta-analysis of R0 resection rate

**Figure 3:**
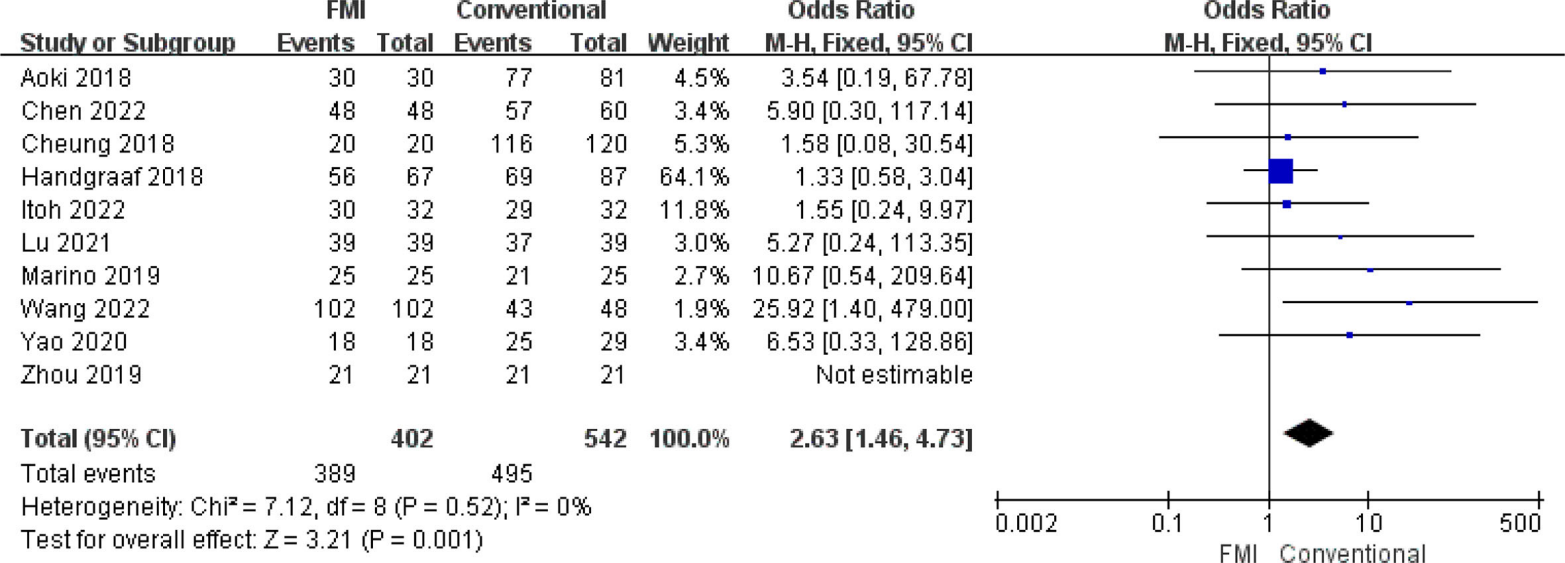
Forest plot on R0 resection rate.

10 studies (21–25, 27, 28, 31, 32, 34) including 944 patients reported the R0 resection rate, there was no heterogeneity between the two groups (*p* = 0.52, *I^2^ =* 0%), and fixed-effects model analysis was used. The meta-analysis results showed that the R0 resection rate of the FMI group was significantly higher than that of the conventional group (OR = 2.63; 95% CI: 1.46~4.73, *p* = 0.001) (**Figure 3**).

#### 3.2.3 Meta-analysis of overall complications

**Figure 4:**
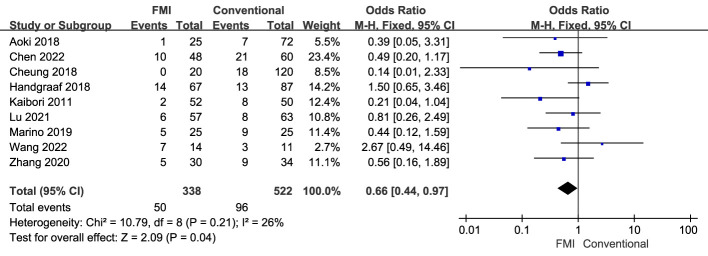
Forest plot on overall complications.

9 studies (21–24, 26–28, 31, 33) including 860 patients reported the overall complications, there was no heterogeneity between the two groups (*p* = 0.21, *I^2^* = 26%), and fixed-effects model analysis was used. The meta-analysis results showed that the overall complications rate of the FMI group was lower than that of the conventional group (OR = 0.66; 95% CI: 0.44~0.97, *p* = 0.04) (**Figure 4**).

#### 3.2.4 Meta-analysis of complications of grade III or above

**Figure 5:**
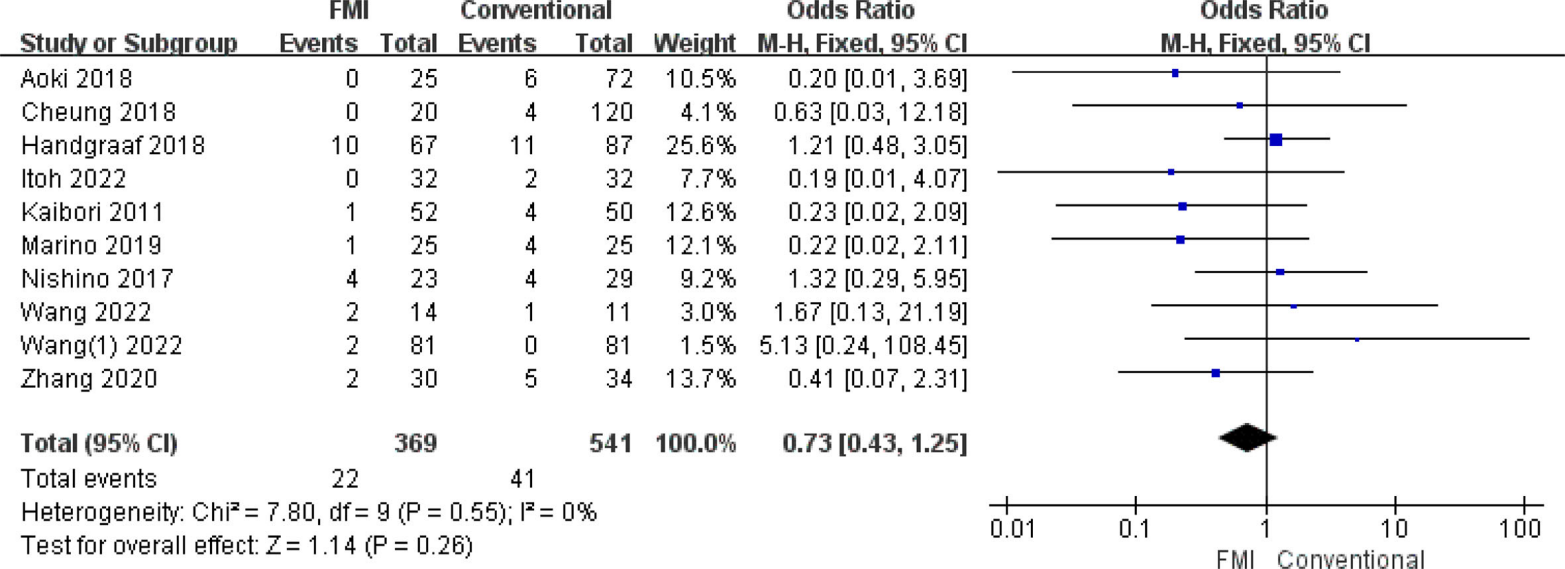
Forest plot on complications of grade III or above.

10 studies (21–26, 28–31, 33) including 910 patients reported the complications of grade III or above, there was no heterogeneity between the two groups too (*p* = 0.55, *I^2^* = 0%), and fixed-effects model analysis was used. The results of meta-analysis showed that there was no significant difference in the complications of grade III rate between the two groups (OR = 0.73; 95% CI: 0.43~1.25, *p* = 0.26) (**Figure 5**).

#### 3.2.5 Meta-analysis of biliary fistula

**Figure 6:**
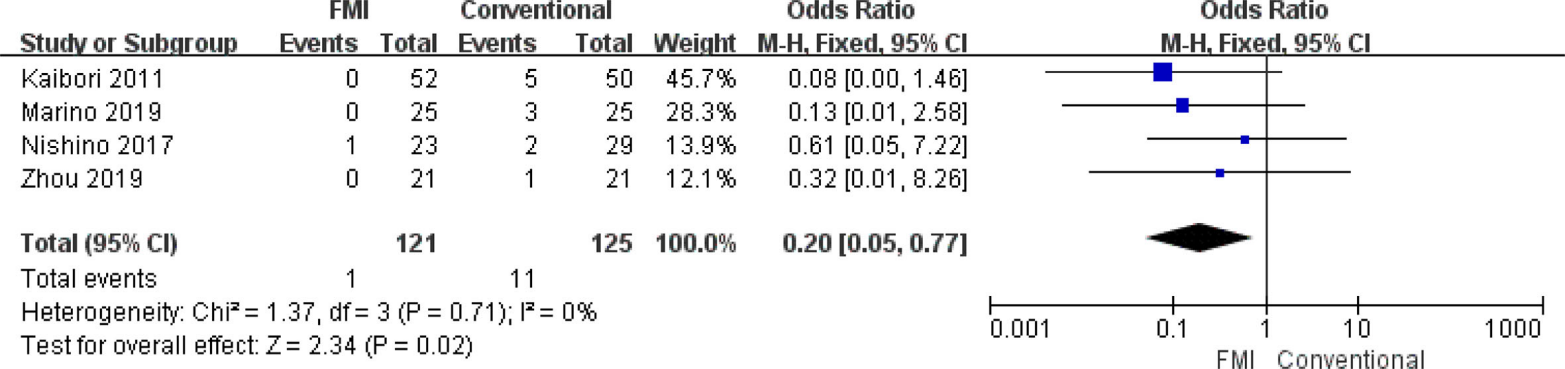
Forest plot on biliary fistula.

4 studies (26, 28, 29, 34) including 246 patients reported the biliary fistula, there was no heterogeneity between the two groups (*p * = 0.71, *I^2^* = 0%), and fixed-effects model analysis was used. The meta-analysis results showed that the biliary fistula rate of the FMI group was significantly lower than that of the conventional group (OR = 0.20; 95% CI: 0.05~0.77, *p* = 0.02) (**Figure 6**).

#### 3.2.6 Meta-analysis of liver failure

**Figure 7:**
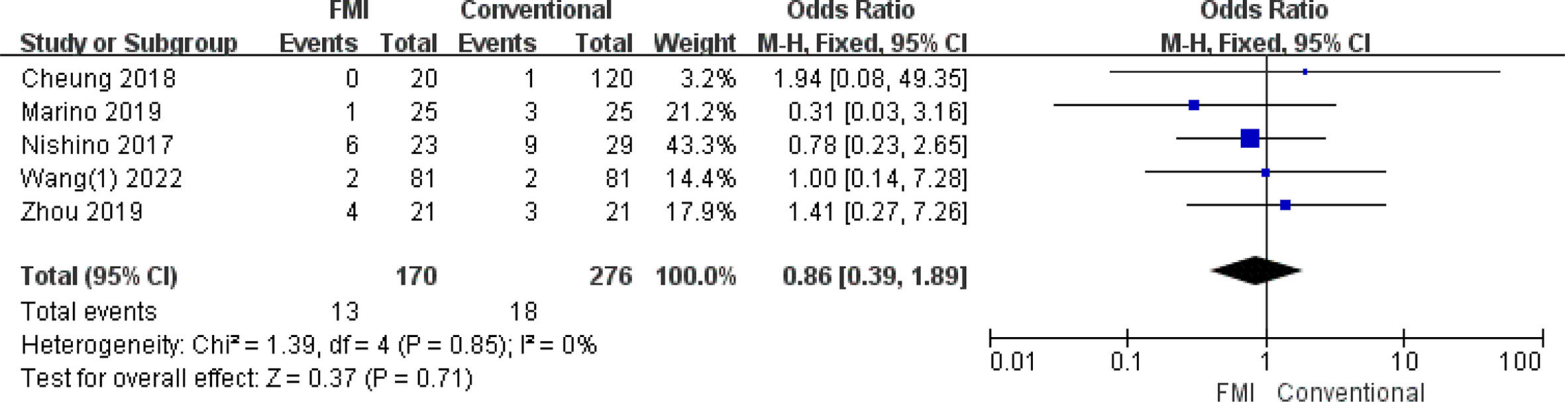
Forest plot on liver failure.

5 studies (23, 28–30, 34) including 446 patients reported the liver failure, there was no heterogeneity between the two groups (*p* = 0.85, *I^2^* = 0%), and fixed-effects model analysis was used. The results of meta-analysis showed that there was no significant difference in the rate of liver failure between the two groups (OR = 0.86; 95% CI: 0.39~1.89, *p* = 0.71) (**Figure 7**).

#### 3.2.7 Meta-analysis of intraoperative blood loss (ml)

**Figure 8:**
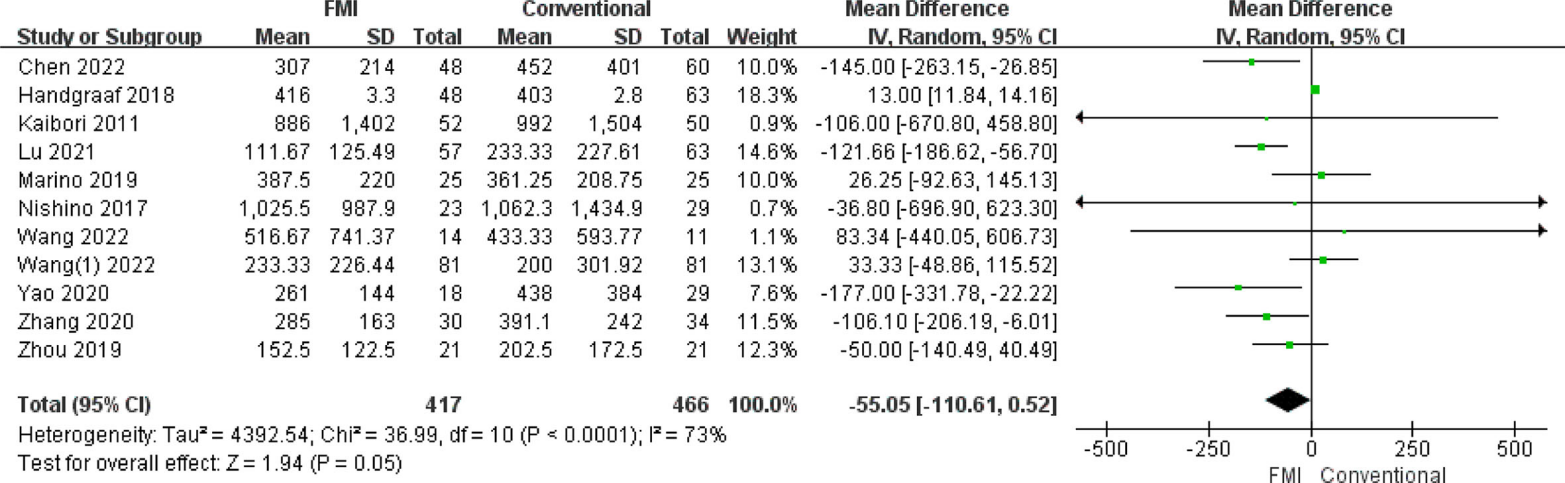
Forest plot on intraoperative blood loss.

11 studies (22, 24, 26–34) including 883 patients reported the intraoperative blood loss. there was highly heterogeneity between the two groups (*p* < 0.0001, *I^2^* = 73%), and a random-effects model analysis was used. The results of meta-analysis showed that there was no significant difference in the intraoperative blood loss between the two groups (MD = -55.05, 95% CI: -110.61 to -0.52, *p* = 0.05) (**Figure 8**). To reduce the heterogeneity, 1 studies (24) were removed (*p* = 0.08, *I^2^* = 42%). The recalculated results show that FMI could reduce intraoperative blood loss (MD = −70.76, 95% CI: −106.11 to −35.41; *p* < 0.000).

#### 3.2.8 Meta-analysis of blood transfusion

**Figure 9:**
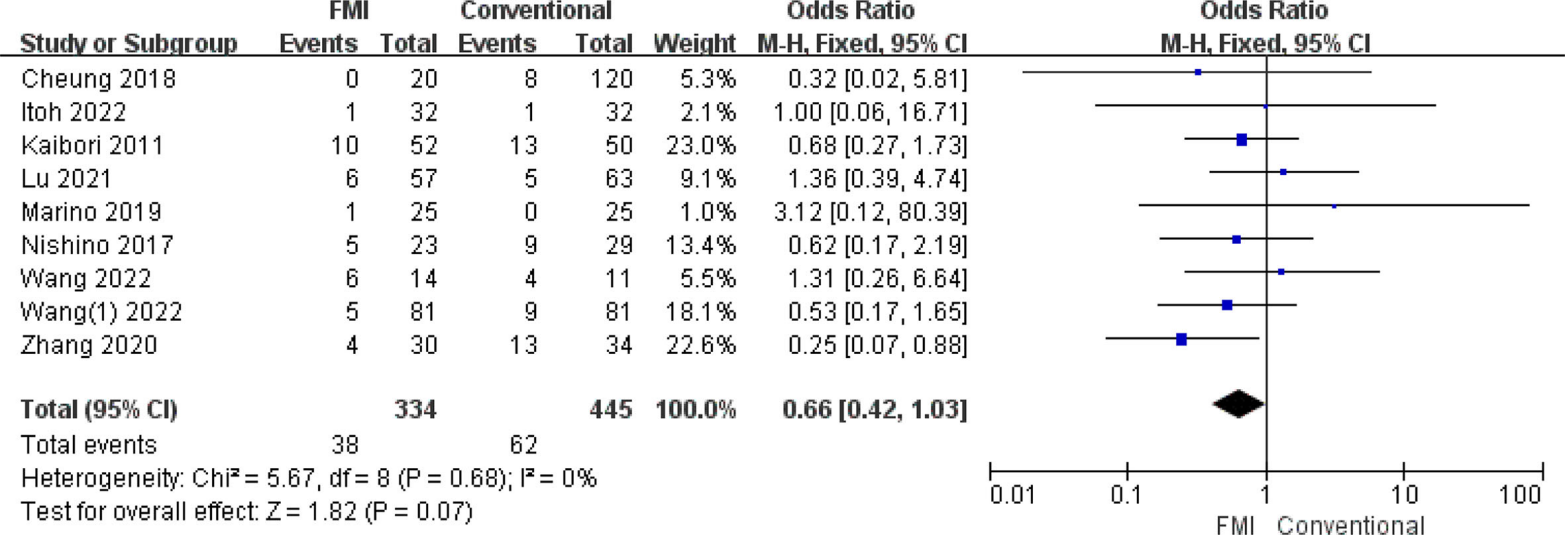
Forest plot on rate of blood transfusion.

9 studies (23, 25–31, 33) including 779 patients reported the rate of blood transfusion, there was no heterogeneity between the two groups (*p* = 0.68, *I^2^* = 0%), and fixed-effects model analysis was used. The results of meta-analysis showed that there was no significant difference in the rate of blood transfusion between the two groups (OR = 0.66; 95% CI: 0.42~1.03, *p* = 0.07) (**Figure 9**).

#### 3.2.9 Meta-analysis of hospital stay (day)

**Figure 10:**
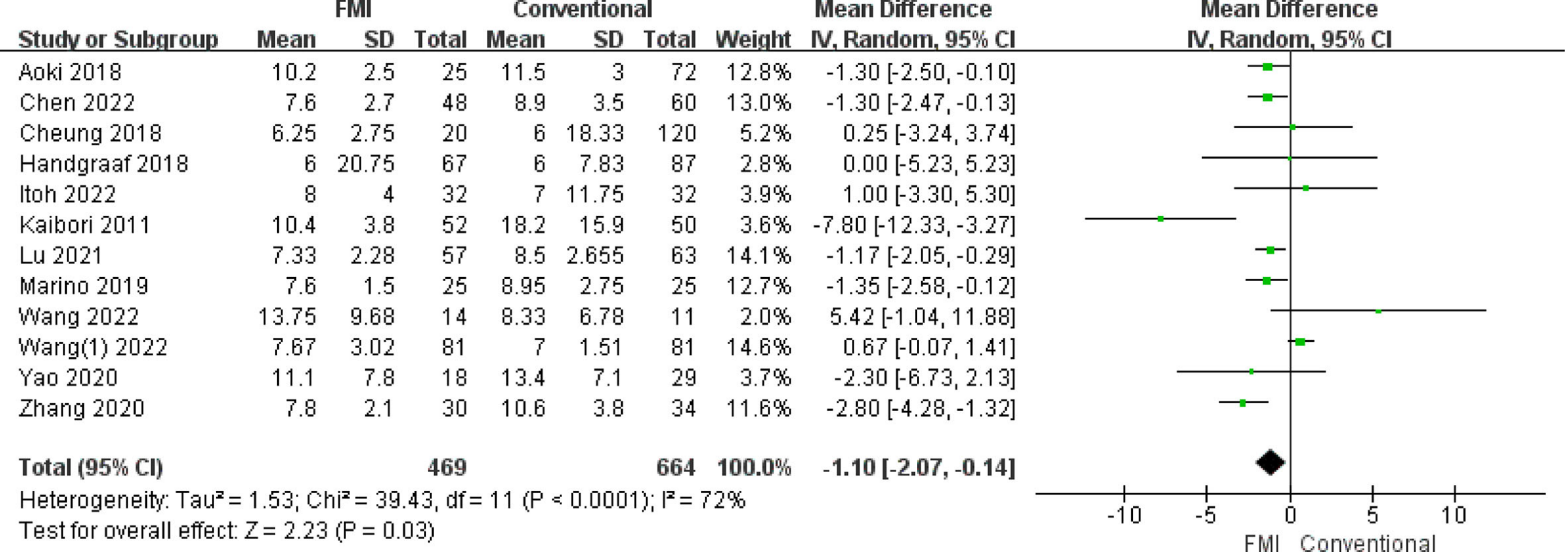
Forest plot on hospital stay.

12 studies (21–28, 30–33) including 1133 patients reported the hospital stay. there was highly heterogeneity between the two groups (*p* < 0.0001, *I^2^* = 72%), and a random-effects model analysis was used. The meta-analysis results showed that the hospital stay of the FMI group was shorter than that of the conventional group (MD = -1.10, 95% CI: -2.07 to -0.14, *p* = 0.03) (**Figure 10**). To reduce the heterogeneity, 1 studies (24) were removed (*p* = 0.05, *I^2^* = 45%). The recalculated results also showed that FMI could significantly shorten the length of hospital stay (MD = −1.41, 95% CI: −1.90 to −0.92; *p* < 0.00001).

### 3.3 Subgroup analysis

Subgroup analyses were further performed based on the surgery type, pathology type, and tumor diameter, yielded similar results to the primary analysis, especially in terms of R0 resection rate. The difference is that the subgroup analysis of laparoscopic hepatectomy showed that the operation time of the FMI group was shorter (MD = −14.84, 95% CI: −27.38 to −2.31; *p* = 0.02), while the analysis of the malignant tumor subgroup showed that there was no difference between the overall complications and intraoperative blood loss, and the analysis of the average tumor diameter < 5cm subgroup showed that there was no difference between the two groups of intraoperative blood loss and hospital stay, but the operation time was shorter in the FMI group (MD = −18.05, 95% CI: −30.96 to −5.13; *p* = 0.006) (**Table 2**).

**Table 2:** Results of subgroup analyses.

Outcomes	No. of studies	No. of Patients	Heterogeneity		The pooled analysis		
			*I^2^*	*p*	HR/OR	95% CI	*p*
laparoscopic hepatectomy							
Operative time	10 (21–23, 25, 27, 28, 30, 31, 33, 34)	872	13%	0.32	−14.84	−27.38, −2.31	0.02
R0 resection rate	8 (21–23, 25, 27, 28, 31, 34)	743	0%	0.73	4.78	1.82, 12.56	0.002
Overall complications	7 (21–23, 27, 28, 31, 33)	604	0%	0.55	0.57	0.35, 0.92	0.02
Complications of grade III or above	7 (21, 23, 25, 28, 30, 31, 33)	602	0%	0.62	0.52	0.23, 1.19	0.12
Intra-operative blood loss	7 (22, 27, 28, 31–34)	571	56%	0.04	-59.58	-118.92, -0.23	0.05
Blood transfusion	7 (23, 25, 27, 28, 30, 31, 33)	625	0%	0.46	0.66	0.37, 1.16	0.15
Hospital stay	9 (21–23, 25, 27, 28, 30, 31, 33)	668	30%	0.19	-1.33	-1.84, -0.83	< 0.00001
Malignant tumor							
Operative time	9 (21–25, 28, 30, 31, 34)	842	21%	0.26	-8.10	-19.53, 3.33	0.17
R0 resection rate	9 (21–25, 28, 31, 32, 34)	866	0%	0.45	2.54	1.39, 4.64	0.002
Overall complications	6 (21–24, 28, 31)	574	39%	0.15	0.73	0.46, 1.18	0.20
Complications of grade III or above	7 (21, 23–25, 28, 30, 31)	692	0%	0.48	0.82	0.43, 1.56	0.54
Intra-operative blood loss	7 (22, 24, 28, 30–32, 34)	545	60%	0.02	-27.93	-82.36, 26.51	0.31
Blood transfusion	5 (23, 25, 28, 30, 31)	441	0%	0.75	0.74	0.34, 1.62	0.45
Hospital stay	9 (21–25, 28, 30–32)	847	58%	0.01	-0.52	-1.51, 0.47	0.31
Average tumor diameter <5cm							
Operative time	7 (21–23, 25, 27, 28, 34)	783	0%	0.58	-18.05	-30.96, -5.13	0.006
R0 resection rate	7 (21–23, 25, 27, 28, 34)	593	0%	0.88	3.50	1.22, 10.00	0.02
Overall complications	5 (21–23, 27, 28)	515	0%	0.80	0.48	0.27, 0.84	0.01
Complications of grade III or above	5 (21, 23, 25, 28, 30)	513	0%	0.48	0.46	0.16, 1.33	0.15
Intra-operative blood loss	5 (22, 27, 28, 30, 34)	482	68%	0.01	-52.51	-124.80, 19.78	0.15
Blood transfusion	5 (23, 25, 27, 28, 30)	536	0%	0.68	0.81	0.39, 1.67	0.57
Hospital stay	7 (21–23, 25, 27, 28, 30)	741	66%	0.007	-0.71	-1.56, 0.14	0.10

### 3.4 Publication bias

**Figure 11:**
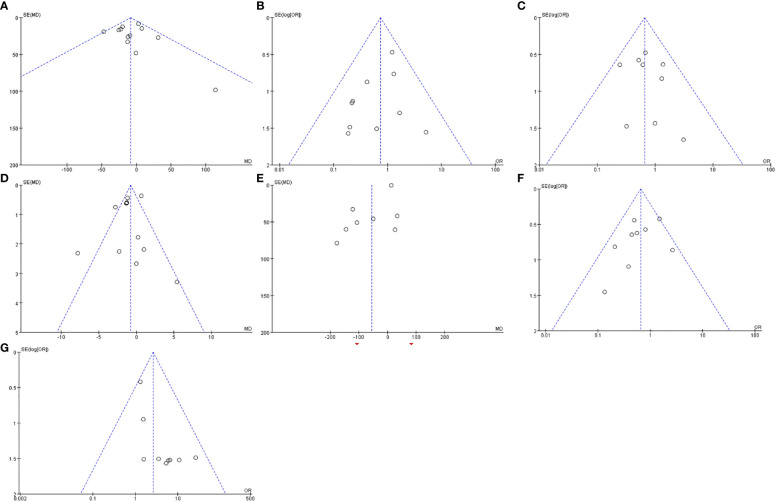
Funnel plots based on operative time **(A)**, complications of grade III or above **(B)**, blood transfusion **(C)**, hospital stay **(D)**, intraoperative blood loss **(E)**, overall complications **(F)** and R0 resection rate **(G)**.

Funnel plots based on operative time, complications of grade III or above, blood transfusion, hospital stay, intraoperative blood loss, overall complications and R0 resection rate are shown in **Figure 11**. The funnel plots of the A to E were not asymmetrical and were evenly vertically distributed, demonstrating no or limited publication bias, but the funnel plots of the F to G show that there is a publication bias.

## 4 Discussion

ICG, a high molecular weight compound, has been widely used clinically to estimate cardiac output and liver function since it was approved by the U.S. Food and Drug Administration (FDA) in 1954. After entering the vein, ICG quickly and is completely bound to plasma protein to form an ICG complex, which is irradiated by a specific near-infrared light source (750 - 810 nm) to emit a fluorescent signal with a peak of about 840 nm. Furthermore, ICG is excreted in an unconjugated form through bile, cannot be cleared by extrahepatic mechanisms, and almost no adverse reactions occur. Based on its safety and the characteristics of fluorescence imaging, ICG-mediated FMI technology has been widely used in the detection of various tumor sentinel lymph nodes (35–37) and perfusion assessment of gastrointestinal anastomosis (38, 39), and its effectiveness has been recognized.

In the field of hepatobiliary surgery, ICG-mediated FMI has been used in surgical procedures. This technique allows the surgeon to visualize a variety of liver tumors (23, 33), bile duct anatomy (40, 41), and hepatic blood flow (42). Surgical resection of liver tumors (primary and metastatic) has been shown to be the cornerstone of treatment with other combined therapies, such as ablation and transcatheter arterial chemo-embolization. Currently, liver tumor surgery is primarily based on three criteria: performing R0 liver resection, maintaining healthy liver tissue as much as possible, and reducing overall complications. R0 resection is the gold standard for any tumor resection, which has been associated with better long-term survival (43). Since Ishizawa, ICG FMI technology has been used several times to resect liver tumors. Compared to intraoperative ultrasound, ICG-mediated FMI technology offers advantages in identifying superficial and tiny tumors. Some published data suggest that the sensitivity of ICG fluorescence imaging to identify superficial liver tumor was 85% (44) to 100% (11). Given the limited penetration, tumor > 5mm below the hepatic capsule could only be identified after resection and sectioning (44), in addition, the false positive rate is as high as 40% (16). And current research always has different perspectives on whether this technology can provide benefits for patients. Therefore, the clinical efficacy of this technology remains controversial. By including a large number of high-quality literacies for meta-analysis, this study analyzes the safety and effectiveness of hepatoma resection guided by ICG-mediated FMI technology, and provides an evidence-based basis for its clinical application.

After strict screening and meticulous analysis, a total of 14 studies were included, involving 1,227 patients who underwent open or laparoscopic hepatectomy, most of whom came from Asia. In addition, the quality scores of the included studies were relatively high, thereby enhancing the credibility of this document. Our analysis results show that the application of ICG-mediated FMI technology in hepatoma resection can improve the clinical results of patients, mainly in increasing the R0 resection rate, reducing the overall complications, the incidence of biliary fistula and intraoperative blood loss, and shortening the hospitalization time, which are related to the characteristics of ICG-mediated FMI. In hepatomatectomy, ICG-mediated FMI technology gives the surgeon significant advantages, helping the surgeon identify all liver tumors from the surface of the liver, and at the same time can guide the tumor resection in real time and immediately evaluate the surgical incision. It is worth noting that the border of ICG fluorescence does not equal the boundary of the tumor, as it is wider than the tumor margin (16, 45), therefore, fluorescence-assisted surgery can improve the R0 resection rate of liver tumors.

ICG fluorescent images are useful for hepatic perfusion, visualization of hepatic segments, and the biliary system. The combination of positive staining and negative staining realizes the visualization of liver segment boundaries, so that the surgeon could break the liver parenchyma in the non-vascular area between the liver segments, preventing large vessel injury in the liver segments, and minimizing intraoperative blood loss. And the ability to visualize the biliary system helps prevent intra-operative bile duct injuries and reduce the incidence of biliary leakage after surgery (26). However, it is undeniable that there are high false positives in ICG fluorescence, e particularly with liver cirrhosis. And given the limited penetration, tumor > 5mm below the liver capsule are hard to detect. Therefore, fluorescence combined with intraoperative ultrasound could significantly improve the detection of liver tumors.

Similar results have been reported in previous studies. In the meta-analysis by Liu et al. (46), 5 RCS and 1 randomized controlled trial (RCT) were included, involving 417 patients who underwent laparoscopic hepatectomy. Their research results show that the application of ICG-mediated FMI technology to laparoscopic hepatectomy can effectively reduce the operative time, blood loss, hospital stay and the incidence of overall complications. In addition, a study by Hu et al. (47) also reported similar results. Compared to these studies, our research advantage is that only patients with liver tumors are included, and the sample size is further expanded, making the research results more credible. In addition, we performed a subgroup analysis depending on the type of surgery, the type of pathology and the diameter of the tumor making the research plan more rigorous.

However, this systematic review has some limitations. First, although the latest research has been included, the number of included studies after strict screening were still relatively small, and all of them are RCS, which may affect the level of evidence in this study. Second, most studies had a small sample size, and few reports on the long-term prognosis of patients, such as the recurrence rate of tumors and the survival rate of patients, which should be compared in future studies.

## 5 Conclusion

In general, our research shows that the application of ICG-mediated FMI technology to hepatoma resection can improve the clinical efficacy of patients, mainly manifested in increasing the R0 resection rate, reducing the overall complications, the incidence of biliary fistula and intraoperative blood loss, and shortening the hospitalization time. However, in view of the shortcomings of fluorescence imaging, combined with intraoperative ultrasound can improve the detection of liver tumors. Of course, considering the limited number of patients included in this meta-analysis, and almost all of the studies included are from Asia, higher quality, large samples, and multicenter RCTs are still needed to verify the reliability of our conclusions.

## Data availability statement

The raw data supporting the conclusions of this article will be made available by the authors, without undue reservation.

## Author contributions

GZ and XQ conceived and designed the study. LZ, ZZ, LY, and SN screened electronic databases. ZW, XZ, and JT extracted data from the selected articles. LZ ZZ, and YP evaluated eligible study quality. Statistical analyses were performed by GZ. GZ and XQ wrote the manuscript. ST and TW supervised the study. All authors contributed to the article and approved the submitted version.
